# Comparative effects of bioelectric and manual toothbrushing on acrylic denture teeth: Surface integrity and wear analysis

**DOI:** 10.1371/journal.pone.0352019

**Published:** 2026-07-02

**Authors:** Luiz A. Pimenta, Carlos Nicolas Nowik, Salar Zeinali, Young Wook Kim

**Affiliations:** 1 Division of Prosthodontics, College of Dental Medicine at Columbia University, New York, United States of America; 2 ProxiHealthcare Advanced Institute for Science and Technology (PAIST), Seoul, Republic of Korea; University of North Carolina at Chapel Hill Davis Library: The University of North Carolina at Chapel Hill Libraries, UNITED STATES OF AMERICA

## Abstract

Bioelectric toothbrushes deliver a low-level microcurrent intended to disrupt biofilm with reduced mechanical force, yet their effects on denture-tooth wear remain unclear. This in vitro study compared surface roughness and material loss of acrylic denture teeth brushed with a bioelectric toothbrush versus a soft-bristle manual toothbrush. Thirty-six heat-cured polymethylmethacrylate (PMMA) maxillary central incisors were randomly allocated to three groups (n = 12): manual brushing, bioelectric brushing without current (“OFF”), and bioelectric brushing with current (“ON”). Specimens underwent 20,000 brushing strokes (≈2 years of home cleaning) under a 200 g load using a brushing simulator in a 1:1 dentifrice/water slurry. Surface roughness (Ra, µm) and specimen weight (g) were recorded before and after brushing, and surface topography was qualitatively assessed by digital microscopy. Baseline roughness did not differ among groups (p > 0.05). After brushing, Ra increased significantly in the manual group (1.538 ± 0.219 to 2.233 ± 0.370 µm) and the bioelectric-OFF group (1.481 ± 0.199 to 2.157 ± 0.403 µm), whereas the bioelectric-ON group showed a smaller, non-significant change (1.547 ± 0.252 to 1.838 ± 0.197 µm); the bioelectric-ON condition resulted in significantly lower final roughness than the other groups (p < 0.05). No significant weight loss was detected in any group (p = 0.71). Microscopy corroborated profilometry, showing fewer and shallower scratches on specimens brushed with the bioelectric toothbrush in the ON mode. Within the limitations of this in vitro model, bioelectric activation was associated with reduced surface roughening of PMMA denture teeth without measurable material loss; however, clinical studies are needed to confirm its relevance under real-world oral conditions.

## Introduction

Electric toothbrushes, powered by rotating or vibrating motors, have improved plaque removal compared with manual brushes and are widely recommended for maintaining oral hygiene [1–4]. At the same time, repeated toothbrushing may contribute to surface abrasion of dental materials and tooth structures, particularly when combined with abrasive dentifrices or excessive brushing force [5]. Such mechanical wear has been associated with the development of non-carious cervical lesions (NCCLs) and progressive surface degradation over time [6–8].

Sonic toothbrushes, which use high-frequency vibrations, are effective at removing plaque and are designed to reduce contact-based abrasion [9,10]. Their mechanism relies on both bristle contact and fluid movement around the tooth surface [11]. However, the literature reports mixed results regarding their long-term effects on tooth surfaces and restorative materials when compared with manual brushing [12–15].

Bioelectric toothbrushes represent a different approach. Instead of relying solely on physical motion, these devices emit low-level electrical currents that alter the permeability of the biofilm’s extracellular matrix. This change disrupts bacterial cell membranes and promotes biofilm detachment through osmotic stress [16–20]. As a result, plaque can be reduced with less dependence on mechanical brushing forces [21].

This mechanism may be particularly relevant for patients who wear removable dentures. Edentulism remains a major global health issue, especially among older adults [22,23]. Denture teeth fabricated from acrylic resin are susceptible to surface wear during routine cleaning procedures, which may compromise their appearance, function, and longevity over time [24–28].

Maintaining adequate hygiene in denture wearers is essential to prevent biofilm-related conditions such as denture stomatitis [28]. Clinical guidelines, therefore, recommend cleaning methods that are both effective in plaque removal and minimally abrasive to prosthetic materials [29]. Technologies that reduce mechanical abrasion while maintaining cleaning efficacy could therefore offer advantages in the maintenance of removable prostheses.

The present study investigates the effect of a bioelectric toothbrush on the surface roughness and material wear of acrylic denture teeth under simulated long-term brushing conditions. The aim was to determine whether this technology represents a less abrasive alternative to manual toothbrushing while maintaining effective cleaning performance.

## Materials and methods

This study did not involve human participants or animals; therefore, institutional ethics approval was not required. Thirty-six prefabricated cross-linked PMMA denture teeth (industrially polymerized) were used to evaluate surface wear and roughness following simulated long-term toothbrushing. The specimens consisted of maxillary right central incisors (Portrait IPN 12G, shade A2; Dentsply Sirona, USA), fabricated using conventional techniques with multilayered interpenetrating polymer network (IPN) polymethylmethacrylate (PMMA). No additional polishing was performed beyond the manufacturer’s standard finish to reflect the as-delivered clinical condition of denture teeth. The teeth were randomly assigned to three groups (n = 12 per group):

**Group 1:** Soft-bristled manual toothbrush (Oral-B Complete Sensitive Toothbrush 35 Extra Soft, USA)

**Group 2:** Bioelectric toothbrush without current (“turned off”) (Great Gums Bioelectric Toothbrush, USA)

**Group 3:** Bioelectric toothbrush with active current (“turned on”) (Great Gums Bioelectric Toothbrush, USA)

### Specimen conditioning

All denture teeth were conditioned at 37 ± 1 °C in a sealed container to equilibrate temperature and moisture prior to testing. (INTBUYING, Zhejiang, China) Each specimen was weighed on an analytical balance (readability = 0.1 mg) at t₀ and again after 24 h. (Crispaire Analytical Balance, China). Conditioning continued until mass stability ≤ 0.1 mg over 24 h was achieved (typically within 48 h), at which point baseline measurements were recorded. The same temperature was used post-brushing before the final weighing to minimize hygroscopic effects. This procedure provides a standardized, physiologically relevant pre-test state for cross-linked PMMA and reduces noise in gravimetric outcomes.

Surface roughness (Ra, µm) was recorded prior to brushing using a contact profilometer (Mitutoyo Surftest SJ-210, Japan) equipped with a diamond stylus (tip radius 5 µm). Three consecutive readings were taken at the same site, each comprising three passes of 0.8 mm at 1 mm/s, for a total analyzed length of 2.4 mm. Measurements were taken perpendicular to the long axis of each tooth. A custom orientation jig ensured consistent positioning of the stylus across repeated measurements. All measurements were performed by the same operator and were not conducted under blinded conditions.

**Fig 1:**
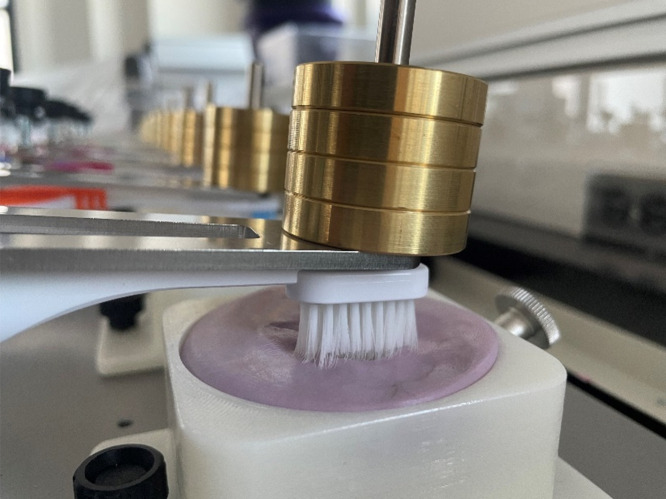
Toothbrush simulator ZM-3.8 with eight specimen chambers.

Following baseline measurements, specimens were mounted in a brushing simulator (ZM-3.8 Toothbrush Simulator; Mechatronic GmbH, Germany) capable of testing eight samples simultaneously (Fig 1). Each specimen was brushed for 20,000 strokes using a linear reciprocating motion (back-and-forth strokes) across the denture tooth surface under a 200 g load, simulating approximately two years of routine cleaning [25,26]. Brushing was performed using a 1:1 slurry of dentifrice in distilled water (Crest Cavity Protection Toothpaste, USA) with a relative dentin abrasivity (RDA) of approximately 95. A conventional toothpaste was selected because surveys show that most denture wearers use regular dentifrices rather than denture-specific products [27]. This choice aimed to simulate real-world practices.

After brushing, the teeth were rinsed under running water, gently dried with tissue, and returned to the oven for 48 hours at 37 ± 1 °C, followed by one hour in the desiccator. This conditioning step was performed to stabilize moisture content and reduce hygroscopic variability of the acrylic resin before gravimetric measurements. Each specimen was then reweighed using the same protocol as before. Surface roughness was reassessed using the profilometer in the same location, using the orientation jig.

Statistical analysis was performed using one-way ANOVA followed by Tukey’s post hoc test (α = 0.05) to assess differences in weight loss between groups. Surface roughness data were analyzed using repeated-measures ANOVA with Tukey’s test (α = 0.05) to compare pre- and post-brushing roughness values and identify differences between groups.

Finally, qualitative surface characterization was performed using a digital microscope (VHX-5000, Keyence Corporation of America, IL, USA) to observe topographic changes and signs of abrasion following the brushing protocol. Representative images were obtained at magnifications ranging from 100× to 200 × , as shown in Fig 3.

## Results

### Surface roughness analysis

Surface profilometry was used to evaluate changes in microroughness on the acrylic denture teeth after simulated toothbrushing. Profilometry measurements were performed under controlled laboratory conditions with stable ambient temperature and humidity. These measurements capture surface features such as peaks and valleys, which can influence plaque accumulation and material wear [30–34]. The mean roughness values (Ra, µm) for each group before and after brushing are summarized in Table 1.

**Table 1:** Mean (± SD) surface roughness (Ra, µm) before and after 20,000 brushing strokes.

Condition	Before Brushing (µm)	After Brushing (µm)
Manual	1.54 ± 0.22 (A)	2.23 ± 0.37 (B)
Bioelectric Effect Off	1.48 ± 0.20 (A)	2.16 ± 0.403 (B)
Bioelectric Effect On	1.55 ± 0.25(A)	1.84 ± 0.19 (A)

*Different capital letters indicate statistically significant differences between groups (p < 0.05).*

Surface roughness data were analyzed using repeated-measures ANOVA to assess within-group changes over time and one-way ANOVA with Tukey’s post hoc test to compare post-brushing roughness among groups. Tukey’s post hoc analysis showed that the specimens brushed with the bioelectric toothbrush in the “ON” mode had significantly lower final roughness compared to both the manual brushing and bioelectric “OFF” groups. No significant differences were observed between the manual and “OFF” groups.

**Fig 2:**
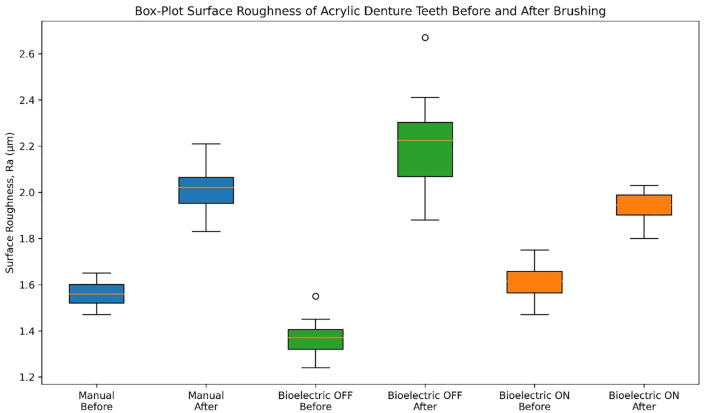
Box plot of surface roughness values by group (blue: Manual; green: Bioelectric OFF; orange: Bioelectric ON).

The bioelectric “ON” group showed lower post-brushing Ra than the manual and “OFF” groups. Fig 2 presents a box plot illustrating the distribution of roughness values across groups.

### Weight loss analysis

To assess material loss, the weight of each specimen was recorded before and after brushing. The results are presented in Table 2.

**Table 2:** Mean and Standard deviation of acrylic teeth weight (in grams) before and after toothbrushing testing.

Condition	Before Brushing (g)	After Brushing (g)
Manual Brushing (Control)	0.39 ± 0.14 (A)	0.39 ± 0.14 (A)
Bioelectric Effect Off	0.35 ± 0.14 (A)	0.35 ± 0.14 (A)
Bioelectric Effect On	0.35 ± 0.14 (A)	0.35 ± 0.14 (A)

*The same capital letters indicate no statistically significant differences between groups (p > 0.05).*

Statistical analysis using one-way ANOVA showed no significant differences in weight change among the groups (p > 0.05). These findings indicate that, despite differences in surface roughness, none of the toothbrushes caused measurable loss of material under the conditions tested.

### Qualitative surface characterization

**Fig 3:**
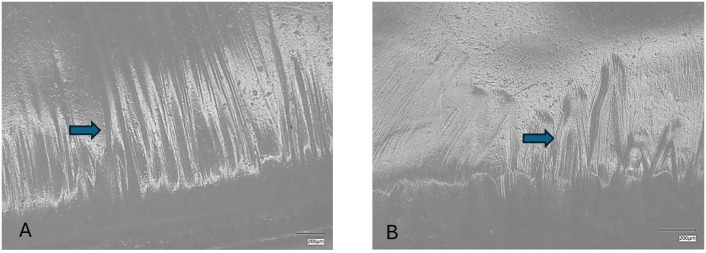
Representative microscopic images of acrylic denture tooth surfaces after 20,000 brushing strokes. **(A)** Soft-bristle manual toothbrush group. **(B)** Bioelectric toothbrush (ON mode) group.

Five representative specimens from each group were examined using a digital microscope (VHX-5000, Keyence) to qualitatively assess surface features following brushing. Representative images were obtained at magnifications ranging from 100× to 200 × , as shown in Fig 3. The microscopy images showed surface features consistent with brushing-related abrasion, including grooves and scratch patterns on the denture tooth surfaces. Specimens from the manual and bioelectric “OFF” groups generally displayed more evident abrasion patterns, whereas surfaces from the bioelectric “ON” group appeared comparatively more uniform. These observations are descriptive and are presented to visually complement the quantitative profilometry findings.

## Discussion

To date, only one commercially available toothbrush, the model evaluated in this study, utilizes a bioelectric mechanism and is registered with the U.S. FDA. While certain electric toothbrushes are marketed with terms such as “ionic” or “bioelectric,” these devices typically do not employ microcurrent technology designed to disrupt plaque at the microbial level. In this context, the term “bioelectric” refers specifically to the use of low-level direct current intended to alter biofilm structure and facilitate plaque disruption through non-mechanical mechanisms. Because this approach differs from conventional brushing technologies, it is important to evaluate how these devices interact with dental materials, particularly acrylic denture teeth, which are susceptible to surface wear during routine cleaning.

This study represents an initial step in evaluating how this technology affects the surface properties of denture teeth. To isolate the role of the electric current, the experimental parameters were standardized, including a single toothbrush model, one dentifrice of moderate abrasivity (RDA ≈ 95), and a single prefabricated cross-linked PMMA acrylic denture tooth material (IPN PMMA). Specimens were evaluated in their manufacturer-finished state without additional polishing, allowing assessment of surface changes under conditions that closely reflect the clinical delivery of denture teeth. While this design strengthens internal validity, future studies should expand these findings by evaluating different resin systems—including CAD/CAM polymers or microfilled composites—as well as various dentifrice formulations and additional toothbrush models using similar or alternative technologies.

The abrasive level of the dentifrice is a primary determinant of three-body abrasion and surface texturing in polymers such as cross-linked PMMA. To isolate the effect of electrical activation, we standardized the toothpaste to Crest Cavity Protection (hydrated silica; RDA ≈ 95) and kept the slurry ratio constant across groups. Using a single RDA enhances internal validity but limits external generalizability to formulations with different abrasivity. In general, higher-RDA pastes are expected to accelerate roughness development and material removal, whereas lower-RDA pastes may better preserve gloss but could reduce stain/plaque removal efficacy [35,36]. Future studies should map response surfaces across multiple RDA levels and abrasive chemistries to determine whether the relative ranking observed here persists under more abrasive or less abrasive conditions.

Our findings suggest that bioelectric activation significantly reduced surface roughness compared to both the manual toothbrush and the same device without electrical current. This difference may be related to changes in bristle–surface interactions during brushing under electrical activation. One possible explanation involves triboelectrification, whereby an applied electric field may influence surface energy and frictional behavior. However, this mechanism was not directly investigated in the present study and should be considered speculative. [37] These changes may reduce the mechanical force exerted by the bristles, resulting in more uniform, less abrasive contact with the denture surface.

A plausible explanation is that the applied current could modulate local bristle–surface interactions (e.g., bristle splay/contact heterogeneity, slurry entrainment) under constant normal load, thereby altering micro-abrasion. This mechanistic interpretation is hypothetical and was not directly measured here; targeted experiments are needed to confirm it.

Surface roughness values exceeding Ra ≈ 0.2 µm have been reported as critical for increased bacterial plaque retention on dental materials [38]. Although differences among brushing conditions were observed in the present study, all post-brushing roughness values remained above this threshold. This finding underscores the clinical importance of minimizing additional surface abrasion in denture teeth, as further roughening may increase susceptibility to biofilm accumulation.

Profilometry results confirmed this effect, with the “Bioelectric ON” condition producing a smaller increase in surface roughness following 20,000 brushing strokes. This was statistically supported by one-way ANOVA and Tukey’s post hoc comparisons. These observations may be related to altered bristle–surface interactions under electrical activation. Although microcurrent-assisted plaque disruption has been proposed in previous studies [39], the present study did not evaluate plaque-removal efficacy, and this interpretation remains hypothetical.

These results carry potential relevance beyond prosthetic materials. Like denture teeth, natural tooth structures, especially exposed dentin or thin cervical enamel, are vulnerable to mechanical abrasion from brushing [40–43]. Although abrasion studies often focus on enamel and dentin, the present findings specifically address the surface effects on PMMA denture teeth [44,45]. While natural tooth structures such as dentin and thin cervical enamel are also susceptible to brushing-related wear, direct comparisons should be made cautiously. The clinical relevance of our results lies in highlighting how bioelectric brushing may help limit surface changes in denture materials, thereby supporting the longevity and hygiene of prosthetic appliances.

Microscopic analysis provided further support for these findings. The group brushed with the bioelectric toothbrush in its activated mode consistently showed smoother surfaces with fewer and shallower scratches. (Fig 3) In contrast, the manual brushing group exhibited irregular and more pronounced abrasions, likely due to inconsistent bristle pressure and motion. These micro-defects are not only a cosmetic concern; they may also reduce the longevity of dental prostheses by facilitating microbial adhesion and mechanical degradation over time. While our study showed differences among groups, the post-brushing roughness values observed still exceeded this threshold, underscoring the clinical importance of minimizing surface abrasion in prosthetic materials.

The smoother finish observed with the “ON” group suggests a more controlled brushing action, likely aided by the microcurrent’s influence on bristle movement or contact pressure. This may help preserve the surface integrity of acrylic materials and, by extension, maintain the functional and esthetic properties of prostheses. While long-term clinical data are needed, these findings align with the goal of reducing cumulative wear on both prosthetic and natural surfaces by using less-abrasive hygiene tools.

From a clinical standpoint, bioelectric toothbrushes may offer a viable alternative for patients prone to overbrushing, with restorations, or who use removable prosthetic appliances. A more uniform, less abrasive brushing interaction may help reduce cumulative surface wear and help preserve the functional lifespan of dental materials. Selecting appropriate oral hygiene tools is particularly important in aging populations or individuals with limited dexterity, where maintaining both cleanliness and material integrity is essential.

Despite its strengths, this study has several limitations that should be considered when interpreting the findings. Measurements were performed by a single operator and were not conducted under blinded conditions, which may introduce measurement bias. The brushing protocol was performed under controlled in vitro conditions and therefore cannot fully replicate the complexities of the oral environment, including salivary flow, temperature fluctuations, and variability in patient brushing techniques. In addition, only one denture tooth material and a single dentifrice were evaluated, which limits the generalizability of the results to other materials and formulations. The mechanism underlying the bioelectric effect was inferred rather than directly measured. Furthermore, the sample size was determined based on feasibility and prior in vitro studies, and no formal statistical power calculation was performed. Finally, although surface roughness and material loss were assessed, the cleaning efficacy of the toothbrushes—such as plaque or biofilm removal—was not evaluated and should be investigated in future studies.

## Conclusion

Within the limitations of this in vitro study, the type of toothbrush used influenced the surface characteristics of acrylic denture teeth. Activation of the bioelectric toothbrush resulted in significantly lower surface roughness compared with manual brushing and brushing without electrical current, without inducing measurable material loss. Although overall wear outcomes were comparable among groups, the reduced roughness observed with bioelectric activation suggests a potential for less abrasive interaction with denture tooth surfaces.

From a clinical perspective, bioelectric toothbrushes may represent a promising alternative for the hygiene of removable prostheses, with potential benefits for preserving surface integrity and material longevity. However, these findings should be interpreted cautiously, and further clinical studies under real-world oral conditions are necessary to confirm their relevance and to understand better how different brushing technologies interact with prosthetic materials.

## Supporting information

S1 Dataset Raw surface roughness (Ra) and weight data collected before and after simulated toothbrushing for all experimental groups. (XLSX)
